# Improvements in Medical System Safety Analytics for Authentic Measure of Vital Signs Using Fault-Tolerant Design Approach

**DOI:** 10.3389/fmedt.2021.666671

**Published:** 2021-08-25

**Authors:** Prasadraju Lakkamraju, Madhu Anumukonda, Shubhajit Roy Chowdhury

**Affiliations:** ^1^Center for Very Large Scale Integration and Embedded Systems Technology, International Institute of Information Technology (IIIT), Hyderabad, India; ^2^School for Computing and Electrical Engineering, Indian Institute of Technology (IIT), Mandi, India

**Keywords:** correlation, electrocardiography, fault-tolerant systems, fault detection, field programmable gate array, multimodal sensor systems, photoplethysmography, safety analytics

## Abstract

The study presents a novel design method that improves system availability using fault-tolerant features in a non-invasive medical diagnostic system. This approach addresses the effective detection of functional faults, improves the uninterruptible system operating period with reduced false alarms, and provides an authentic measure of vital cardiac signs using diverse multimodal sensing elements like the photoplethysmogram (PPG) and the ECG. Most systems rely on a 1oo1 (one-out-of-one) design method, which inherently limits accuracy in existing practice. In this proposed approach, the quality of segregated authentic vital sign measured values could tremendously benefit the performance of resourceful nursing with negligible alarm fatigue and predict illness more accurately. The system builds upon the selected 2oo2 (two-out-of-two) safety-related design architecture and is evaluated with implemented functions like the fault detection and identification logic, the correlation coefficient-based safety function, and the fault-tolerant safe degradation switching mechanism for accurate measurements. The system was tested on 50 adults of various age groups. The analyzed captured data showed highly accurate vital sign data in this fault-tolerant approach with reduced false alarms. The proposed design method evaluated safety-related mechanisms along with a combination of the same and diverse sensors in a medical monitoring device, showing more reliable functioning of the system and authentic data for better nursing. This design approach showed a 45–55% increased improvement in system availability, thus allowing for accurate and uninterruptable tracking of vital signs for better nursing during critical times in the ICU.

## Introduction

Advanced smart medical system engineering for safety-critical medical applications requires the consideration of several dependable features, such as the functional safety, availability, and reliability of the engineered system in signal processing algorithms (1, 2). With this consideration, the advancement in high-performance electronics and sensors can become affordable and, thus, increase the usage of these smart medical systems in clinical environments, such as performing critical patient treatments like robotic surgeries and medication supervisions through the continuous monitoring of patients (3–5). These smart, intelligent multimodal computational electronics, along with system portability, come with a significant increase in system complexity and bring in major challenges like functional safety, reliability of measurements, and patient safety. This type of safety-critical smart medical device is often subject to an insignificant number of failures with potentially catastrophic impacts on patients. A previous study (6) on medical device recalls between 2006 and 2011 shows a 69.8% increase in the number of products being recalled and a 103.3% increase in the number of adverse events to patients like incorrect medications and deaths, where the majority of the recalls were due to software faults. In a recent report (7, 8) for the year 2018, a significant spike of 126% in product recalls informed the U.S. Food and Drug Administration (FDA) that most of the causes were due to software faults. These detected spurious faults always interrupt the intended system functionality and may generate safe alarms during critical periods. However, the cause of numerous spurious alarms, incorrect measurements of vital signs, single point of failure (SPOF), and undetected faults may cause dangerous effects on patients in nursing care. In the past, some of the medical system designs were adopted with safety feature improvements like data fusion and voting techniques (9–14). However, all these techniques have their limitations and lack the mitigation of challenges, such as the reduction of false alarms by effectively detecting the actual faults, improvements in fault tolerability having built-in safe degradable mechanisms, and providing authentic measurements through the safe computation of vital signs without interruption to system operability.

This study focused on presenting a safety-related, fault-tolerant design approach to improve the safety features in addressing the challenges related to the detection of software faults in a non-invasive medical device. The safety features include: (1) effective fault detection function and (2) fault-tolerability with a safe degradable function. These two functions were implemented and evaluated using the proposed conceptualized 2oo2 (two-out-of-two) safety-related design architecture. This approach provided uninterruptable operability of the system by removing its faults. A detailed framework proposed with five configurable conceptualized safety design architectures based on composite fail-safety techniques were realized and evaluated. A safe degradable function was implemented using Karl Pearson's coefficient of correlation method. Thus, the results were analyzed and evaluated toward the aims of reducing alarms for better nursing with reduced alarm fatigue and providing improved authentic vital signs measured values for better predictability of illnesses and proper assessment of the condition of patients.

The systematic design assurance guidelines (15–17) were followed in the implementation of the proposed architectures. Detailed experimental research activity was performed to evaluate the results and analyzed in detail for safety improvements. The conceptual safety-related 2oo2 design architecture was reused from previous studies (1) in implementing this proposed conceptual fault-tolerant safety architecture for system functional safety evaluation. This study focused on evaluating vital cardiac signs, such as heart rate (HR), using the proposed design approach. The assessment included the continuity of the measurement of HR-values and the authenticity of the measured values. Two diverse sensors were selected to detect biomedical signals, with these signals being based on the physical media of light and electric potential. These sensors, which consist of light-emitting diodes (LEDs) and an optical detector, were used to detect the photoplethysmogram (PPG) biomedical signal, and an electric-potential integrated circuit (EPIC) sensor was used to detect the ECG biomedical signal. Two diverse independent algorithms were used to measure the HR in two independent channels in a 2oo2 safety-related design architecture. Analytics were performed on the captured data to detect and identify the potential systemic faults during vital sign parameter measurement at each independent channel.

A set of measured parametric data was collected periodically, such as HR, from two independent channels and correlated to check for any computational faults. Karl Pearson's coefficient of correlation method was used (1) for safe voting logic and implemented to detect the computational faults of the two independent channels. A built-in test (BIT) fault-tolerant safe degradation function was implemented to identify and isolate systemic and computational faults. A set of sequence operations were performed for any fault detected, such as (1) the system operated in safe mode with safe degradation switching from 2oo2 to 1oo2 or 1oo1 (one-out-of-one) and vice versa toward the isolation of the fault and, thus, provided authentic data; (2) the system used negation error codes for each fault category, thus generating the related alarm for each significant detected fault, and subsequently logging the results. Similarly, the experiment was repeated on the proposed conceptualized five design configurations and evaluated for system resilience. The proposed design configurations are the following: (1) using diverse ECG and PPG sensors and algorithms at each independent channel to measure the HR, (2) using different ECG sensors at each independent channel with diverse algorithms to measure the HR, (3) using different PPG sensors at each independent channel with diverse algorithms to measure the HR, (4) using a single ECG sensor and measuring the HR with diverse algorithms at each independent channel, and (5) using a single PPG sensor and measuring the HR with diverse algorithms at each independent channel. A detailed analysis was carried out for each implemented conceptual architecture, and the rightness of the cardiac vital sign heartrate measurements and the coefficient of correlative results of vital sign measurements between two channels were analyzed and presented using Bland–Altman and correlative plots (18, 19). The recorded data, failure detected signal, and vital sign HR measurements at each channel output and the safe function output results were analyzed for the effective functioning of fault isolation and reducing of a SPOF.

The contributions of this research analysis to this configured medical system prototype, with the analytics on data collected using safety-related design approaches having interfaces with diverse PPG and ECG sensors signified:

1. Improvements in the elimination of PPG and ECG sensor-related problems in bio-signal detection and identification of the root causes for removing the deficiencies in signal processing techniques to extract the authentic vital sign signal information;
2. Efficient predictability in the estimation of illness using accurate data, which eventually improves proper nursing;
3. Improvement in system operability with significantly less insignificant alarms, thus improving the fault detection mechanism, fault identification mechanism, fault tolerability, and safety integrity level of the system for usage in safety-critical applications.

Section Theoretical Framework provides the details about the framework to address the specified challenges in the software faults. This framework includes detailed requirements about the conceptualized architectures and their approaches toward the analysis of the challenges. Section Methodology provides a detailed methodology about the system overview and its realization of two independent channels for experimental studies. Section Experimental Results and Discussion provides the experimental results and analysis about the measured HR-values evaluated along with the fault alarms between the diverse channels and calculated correlation coefficient values for the safety logic. This section tabulated a cause-and-effect system analysis, emphasizing authentic measures, and safety improvements in the medical system.

## Theoretical Framework

Work in safety-related electronic systems design and development is multi-dimensional, which means several safety aspects need to be considered in all phases of the product development life-cycle (PDLC) (15–17). These related systems should adhere to the standard development processes and guidelines and need compliance to respective standards like IEC, ANSI, AAMI, CENELEC, RTCA-DO (15, 17) to domains such as the automotive, medical, railways, and aerospace domains. The objective of any safety-related electronic system should efficiently detect faults. It shall drive the system into a fail-safe mode of system operation based on the severity level of the fault that occurs. The systems where faults and failures like random failures, systematic failures, hardware failures, software failures, or any unknown erroneous errors lead to hazardous situations like death, injury, or environmental damage. However, how effective a system is engineered for a particular application and a signal processing algorithm plays a critical role, as it extracts and provides vital information to make decisions. Generally, for this computation development process, defining the system requirements always challenges dependencies and limitations in sensors, hardware, and other operational environmental factors. In particular, electronic medical systems, sensors, and related computational systems provide important vital sign information regarding the nursing of a subject by monitoring and controlling treatment without any faulty or insignificant information. Thus, the system should not go into a non-operational mode during critical surgical procedures in the ICU.

### Study of Potential Faults With PPG and ECG Sensor Systems

Recent studies have shown significant differences in the many brands of patient monitoring systems, particularly regarding their bias and precision, even though they use identical hardware. These variances are almost certainly due to the different algorithms used in processing the PPG signal (20, 21). Equally, different algorithms were used (21) in processing the EEG signal to mitigate differences in the sensing of materials used and the artifact of interest in the signal to be measured. In-depth studies show that potential faults can notice if anything is compromised or missed in defining the system requirements.

In defining the signal processing requirements, the consideration of a few challenging areas (20) in the PPG signal processing include (1) the selection of LEDs and detectors frequency ranges, (2) the placement of the sensor probes at the fingertips, ear, nose, or forehead, (3) non-invasive probing mechanism, either through light radiation transmission or reflection, (4) other considerable effects like changes in saturation, signal quality, effects of dyshemoglobins, dyes, other pigments, and extraneous factors, and (5) subject physical motion and environmental effects. Equally, in the processing of the ECG signal, the challenging areas (21–24) include: (1) the sensing materials, (2) noise removal and signal quality, (3) non-invasive probing mechanisms using or not using dielectric mediums between the probes and subjects, and (4) other considerable effects like subject physical motion and environmental effects. However, considering these defined requirements and the implementation of the signal processing algorithms software, there is any inconsistency between the algorithms to extract the same vital sign parameter due to faults or limitations at a certain level. In the recent past, few experimental studies with varied sample parameter voting and data fusion methods were used (9, 12–14, 25–29) to better the safety of these devices. Additionally, few studies (14, 21, 22) reported that the same vital parametric data, like HR, can be realized with the different mediums of the sensor.

The present research focused on the fault-tolerant design approach and a safety aspect in the effective detection of the software computing faults in an algorithmic function, which used a defined framework and provided accurate measured parameter data with reduced alarms. The presented framework provided the fault detection and analysis approach and the implementation results using the configurable safety-related 2oo2 design architecture.

### Proposed Conceptual Safety Architectures

The proposed 2oo2 evaluated safety-related design approach (1) was used for further experimental investigations and performed functional safety assessments to validate the implemented bio-signal processing functions in safety-critical medical applications of the biomedical systems. The proposed concept evaluated a set of configurable diverse medical sensors and different signal processing algorithms to measure selected vital parameters. This approach of processing the same parameter in a diverse method, with correlative analytics, offered a scope to improve a useful technique in detecting faults. It also provided options to implement fail-safe degradation mechanisms, and the related usage of safety-related design architecture provided redundancy and availability.

This study considered five different concepts for analysis by configuring the system with diverse ECG and PPG sensors and diverse algorithmic computing software. A correlative analysis was performed and logged the results for each configuration. It tabulated all analysis inferences for each detected abnormality in the functional software and the results with analysis of cause and effects toward improving functional safety. The functional description of the 2oo2 architecture including hardware, software, sensors, and its algorithms used is detailed (1). The set of five selected configurations for framework analysis is described below.

#### Multimodal Sensor Configurable 2oo2 System

**Figure 1:**
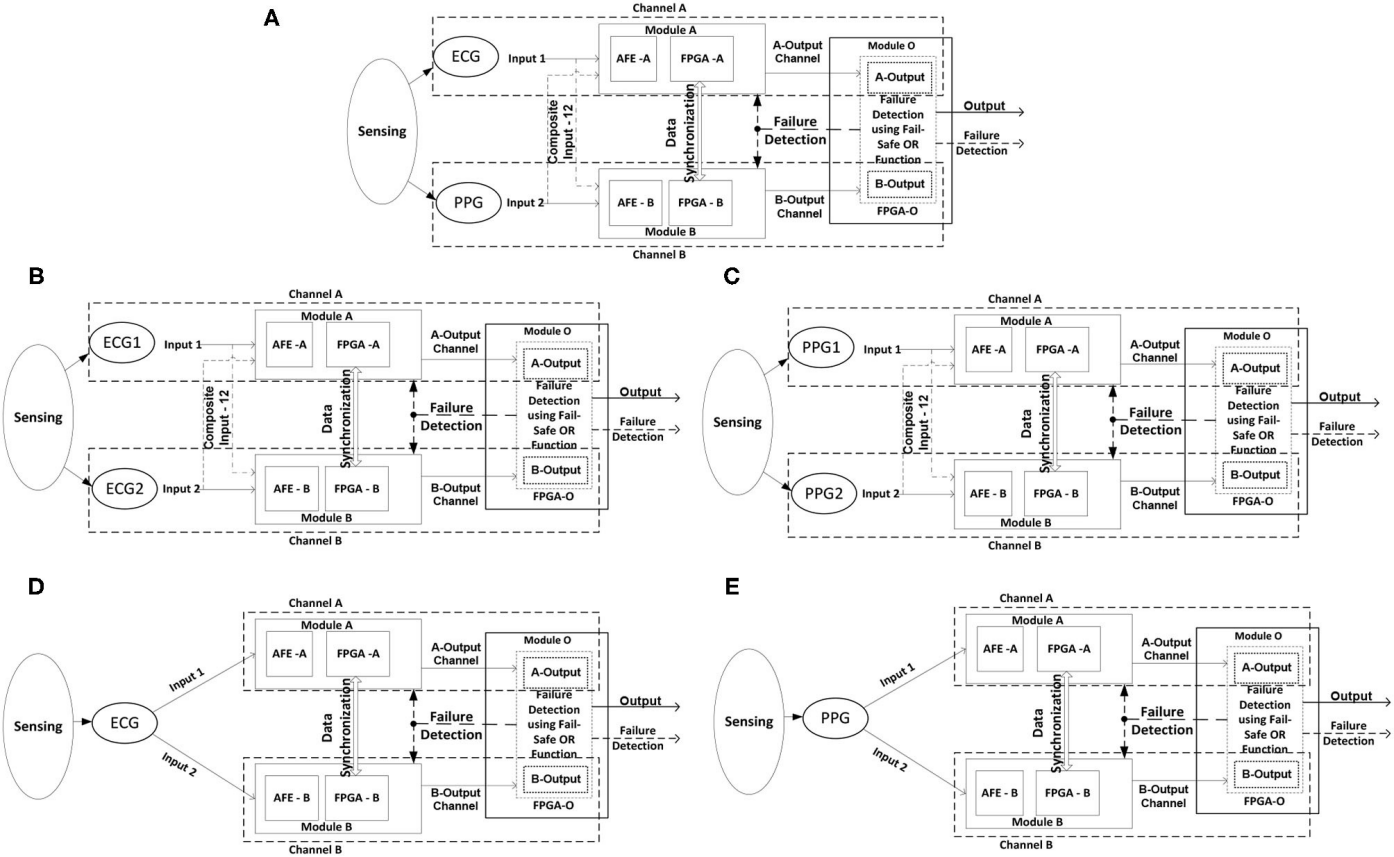
Configurable 2oo2 system. **(A)** ECG and PPG sensor and diverse algorithm. **(B)** ECG1 and ECG2 sensor and the same algorithm. **(C)** PPG1 and PPG2 sensor and the same algorithm. **(D)** Single ECG sensor and the same algorithm. **(E)** Single PPG sensor and the same algorithm.

The 2oo2 System in Figure 1A is configured in 2oo2 with diverse sensor inputs, i.e., the ECG sensor interfaced to the analog front-end (AFE) device in channel-A and the PPG sensor interfaced to the AFE integrated chip (IC) in channel-B. Both channels are configured with selected diverse algorithms (1) to process the HR parameter. The outputs of modules A and B from each channel correlated with a “safe correlative-bounded configurable limits” function are implemented in Module-O to detect faults and generate the failure detection signal. An output signal consisted of accurate parametric data along with processed alarm signal data generated by the fault-tolerant safe degradation function. The safe function implementation included Karl Pearson's coefficient of correlation method, which used (1) a time series sliding window technique between the data of both channels and a fault-tolerant safe degradation voting logic function for reliable switching between the computed output of the channels.

As part of the framework analysis, the recorded output data were analyzed toward the reliability and efficiency of the implemented algorithmic software function and provided the inferences on improvements in the system level requirements. Thus, a practical HR computing function was realized in both channels with authentic HR output with reduced alarms. Similarly, this analysis was further carried out on selected concepts to improve the safety check, like:

a) System configured with diverse ECG or PPG sensors with the same algorithms in both channels, as shown in Figures 1B,C, providing the opportunity to investigate the sensor sensitivity and related deviances in the functional requirement for measuring the same desired parameter.
b) A system configured with one sensor interfacing with both channels, with the same algorithmic function for computation, as shown in Figures 1D,E. This provided the opportunity to investigate the response of the safety functions to implemented improvements in defining the requirements. Furthermore, it helps in the evaluation of system-level hardware failure analysis.

### Evaluation Framework

Safety compliance is a systematic process followed in every phase of the product development life cycle. Every safety-related electronic system aims to detect faults when the system is in an active state and shall drive the system into a fail-safe state. The safe state shall be defined based on the mode of system operation in the active state. During the system operation at a specific mode, the cause of faults and failures is categorized as negation codes. It needs to be defined by labeling and the severity level of the fault that occurs.

In this study, we focused on the functional safety validity of a specific signal processing algorithmic software function and explained the implemented fault detection logic in the 2oo2 approach, its theoretical fault identification mechanism, and its further evaluation framework.

#### 2oo2-Fault Detection Logic and Analysis

**Figure 2:**
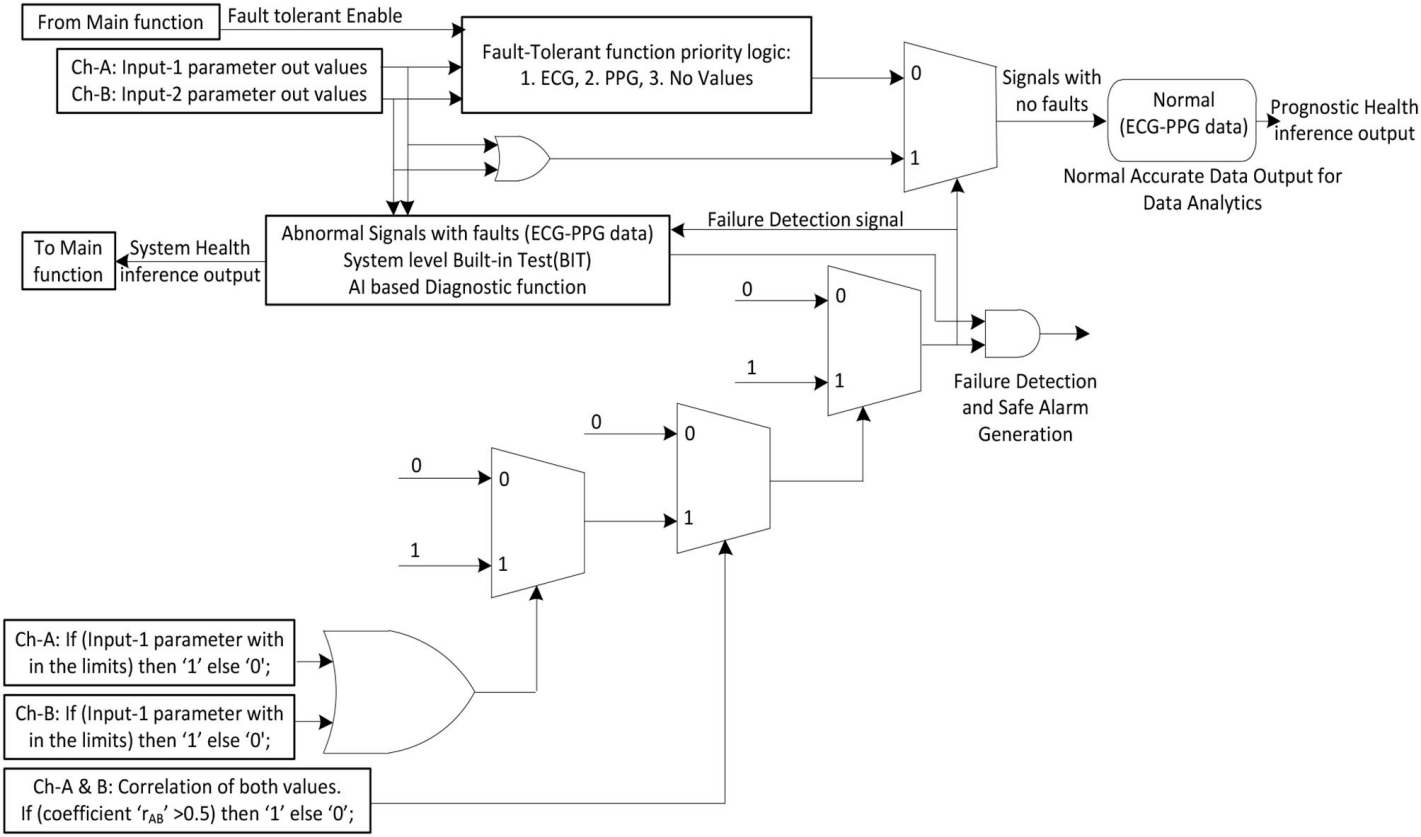
2oo2 fault detection and fault-tolerant logic implementation.

The function 2oo2 fault detection logic received the parameter input data from channel-A or channel-B and conditionally checked between the threshold limits of that particular parameter. The generated outputs were logically ORed as a parallel circuit, and the hardcoded safe-output was selected using the correlative coefficient condition (>0.5), which has a moderate- to a very high-state relationship with parameter “*r*” (1), as shown in Figure 2, to generate the fault detection signal. Furthermore, this fault detection signal was driven back as a feedback input to the primary function, which triggered the diagnostic BIT functions to generate the output enable signal. As shown, the primary function triggered a fault-tolerant enable signal for every authentic fault detected. Thus, a predefined priority-based fault-tolerant safe degradation sequence was initiated to select the apt output to pass onto the display. However, if there is no fault detected, both channel output parametric values were in a good positive relationship. Thus, a selected ORed output was released to the display and AI-based computed prognostic health inference outputs.

**Figure 3:**
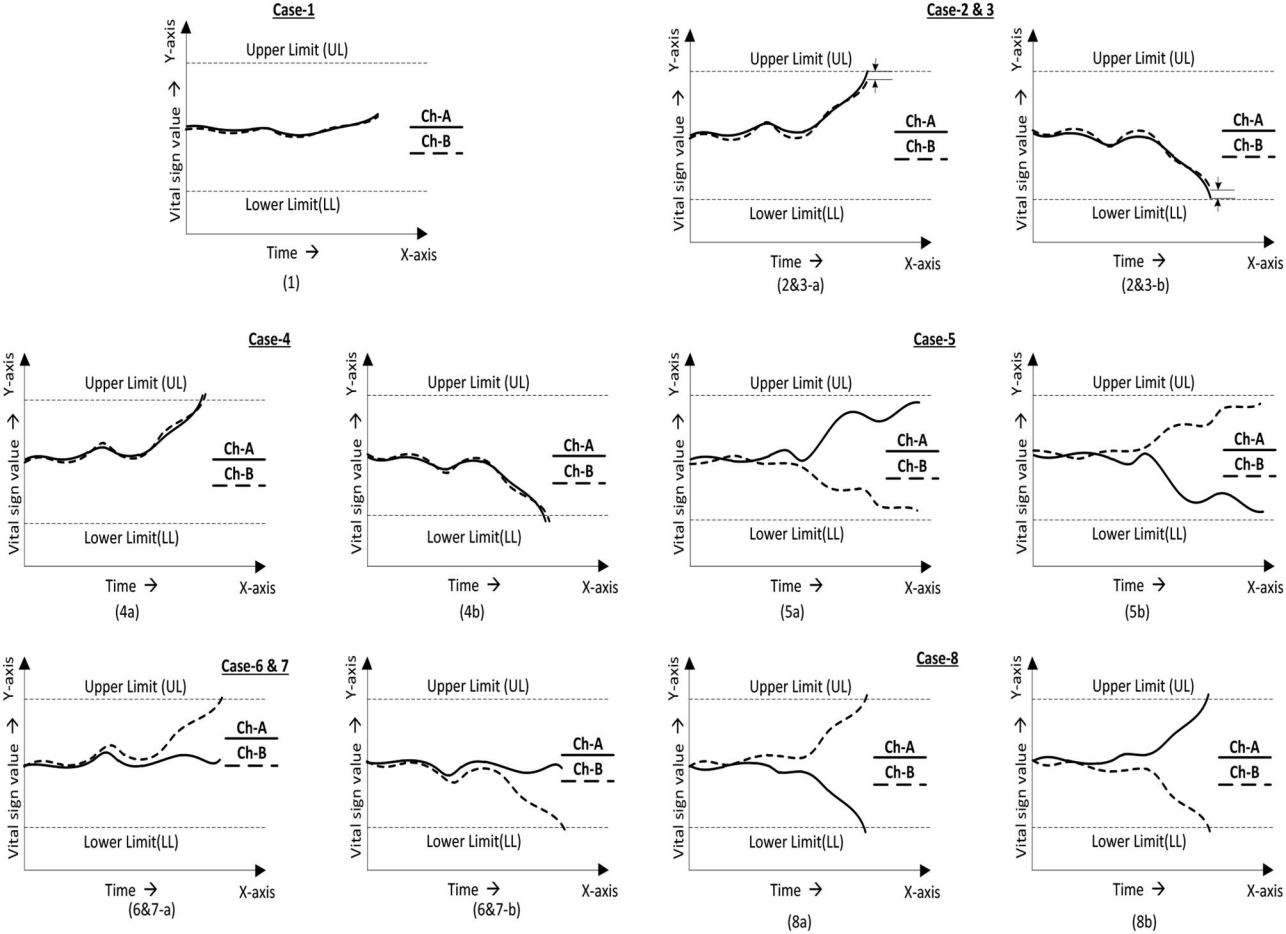
2oo2 voting logic cases for assessment. (1) Values are in a relationship and are in the normal range. (2 and 3) Values in relationship with one signal drift toward the limit. (4) Values in relationship with both signals drift toward the limit. (5) Values out of a relationship and in the normal range. (6 and 7) Values out of a relationship with one signal drift toward the limit. (8) Values out of a relationship with both signals drift toward limits.

In the process of the fault identification mechanism, a defined (1) positive correlation coefficient constant value was compared and continuously monitored to a correlation coefficient value measured between two independently received output-parameter values. Thus, a signal was generated when there was deviance in the relationship between them. In theory, as per the 2oo2 safety-related voting approach, both channels needed to fail to initiate the system to fail-safe mode. Thus, a failure detection signal would then be generated when both the relationship signal and ORed output signal failed. All identified cases were analyzed as detailed in Figure 3 from one to eight cases to determine the actual fault. As part of the evaluation framework, all these cases are tabulated in Tables 1, **3** to analyze a selected vital parameter and were evaluated by checking the validity of the related implemented function for safety.

**Table 1:** 2oo2-based safe-voting logical truth table for fault detection using a correlative technique.

**Evaluation cases**	**Ch_**A**_**	**Ch_**B**_**	**r_**AB**_ (>0.5)** ** (Correlation coefficient)**	**Output signal** ** (2oo2 fault-tolerant)** ** (O_**AB**_)**	**Fault detection** ** (Diagnostics) Yes/No** ** (Fd)**
Case-1	True	True	True	No alarm	No
Case-2	False	True	True	No alarm	Yes
Case-3	True	False	True	No alarm	Yes
Case-4	False	False	True	Alarm	No
Case-5	True	True	False	Alarm	Yes
Case-6	False	True	False	Alarm	Yes
Case-7	True	False	False	Alarm	Yes
Case-8	False	False	False	Alarm	Yes

Following standard compliance, best development practices, and analysis framework approaches in implementing safety-critical medical systems significantly addressed the identified challenges and improved the safety features of the medical system. As part of the evaluation framework, a detailed analysis was carried out for a specific vital parameter. Preliminary analytical research results showed improvements in functional safety in the reduction of spurious alarms, effective detection of functional faults, and improvement of the uninterruptable function of the system by providing authentic measurements.

## Methodology

Experimental research activities of this part of the project were divided into three main phases. The first phase was dedicated to the study and evaluation of the aptness for use of the safety-related architectures in the targeted non-invasive diagnostic medical monitoring and control systems to measure basic vital parameters and identify suitable sensors for sensing biomedical signals like sensing through electric potential, sound, and light. The second phase was dedicated to the design and realization of the three-independent modular designed channels. Each channel was interfaced with a suitable sensor and each channel module based on a field-programmable gate array (FPGA) design with a modular integrated system interface research prototype was built for experimental research studies. The final phase was the validation of the experimental research platform. The system was evaluated with varied configurations with sensors, configurable safety-related architectures, and FPGA circuits. Lab and field trials were performed to assess vital parameters like HR, address the mentioned challenges, reduce fault alarms, identify algorithm limitations, and improve uninterruptable functionality with safe degradation mechanisms.

The present study focused on the final phase of the research activities to improve fault detection mechanisms and, subsequently, address the mentioned challenges. Using configurable safety-related architectures with a combination of ECG and PPG sensor interfaces, the details of the first and second part of the research prototype were used (1) to build activities, design evaluation procedures, and application protocols.

### System Overview

**Figure 4:**
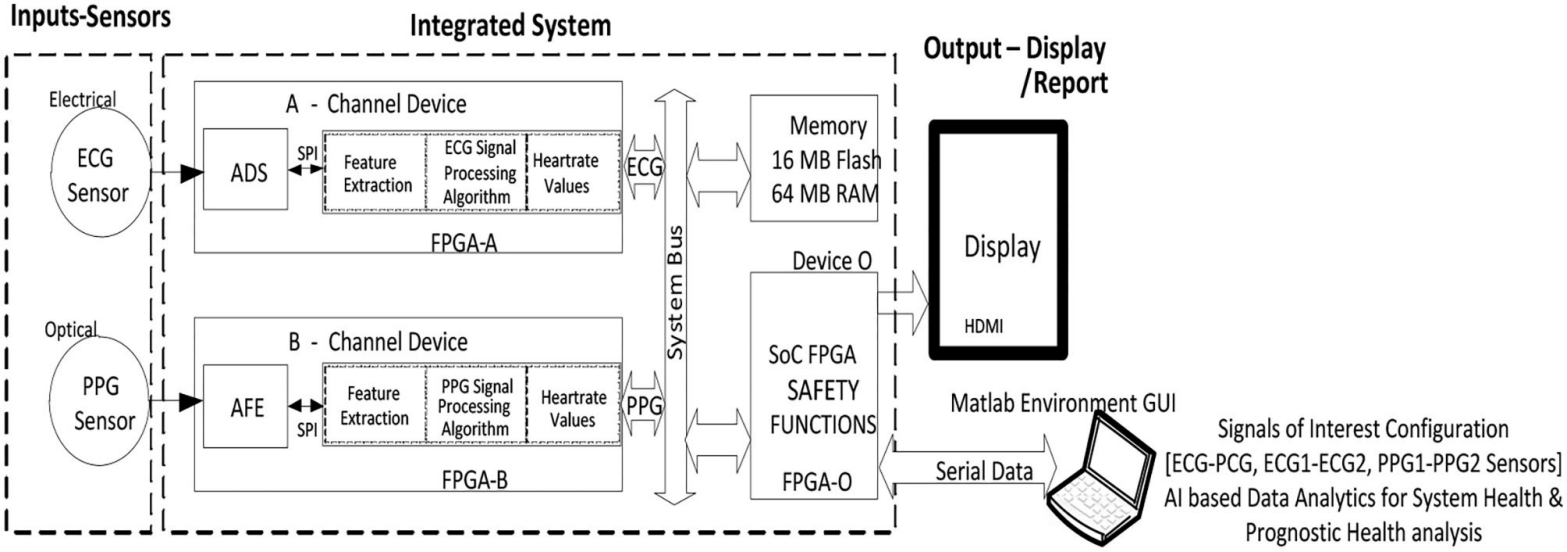
2oo2 fault-tolerant cardiac health monitoring system block diagram.

The cardiac health monitoring system (CHMS) is modular and configured to a 2oo2 safety-related computing platform. The system consists of two independent operating channels, as shown in Figure 4 and explained in (1) detail with CHMS Graphical User Interface (GUI) interfaces for test evaluations.

### Sensors—PPG, ECG, and Its System Configurations

The set of PPG sensors (LEDs and a detector), ECG sensors (EPIC and Ag-AgCl electrodes), and its interfaces used in this system configuration are detailed (1). These combinations of sensors bonded into a single module for convenience, and each module was placed on a subject at the prescribed location for better measurements. These sensor modules were packaged in modular combinations like ECG–PPG, ECG–ECG, and PPG–PPG, and each module was interfaced with the integrated computing system, as shown in Figure 4, configured, and evaluated for each conceptual design.

### 2oo2 Based Safe-Voting Method Using Correlation Analytics

**Figure 5:**
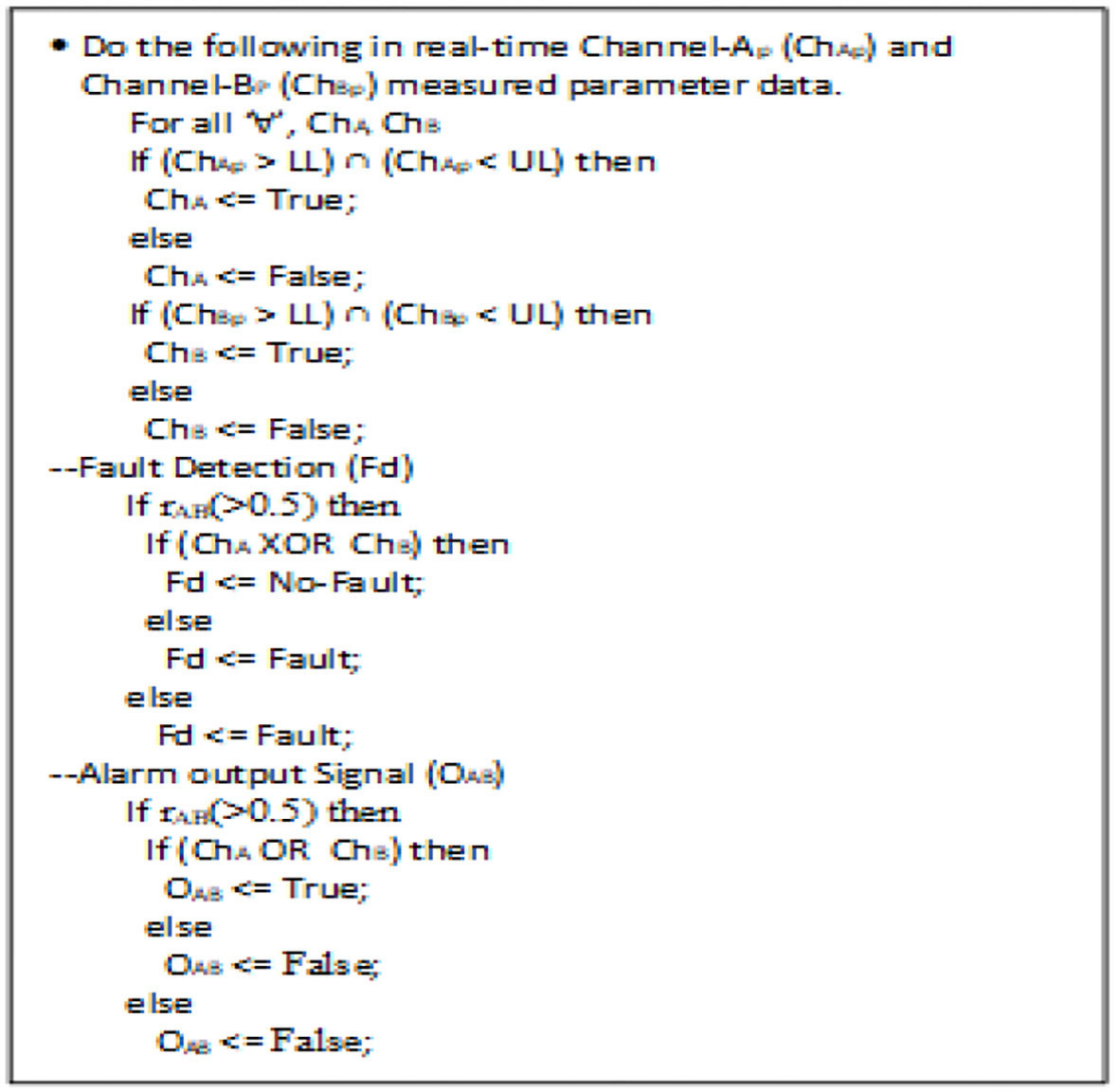
Pseudocode for fault detection analytics and alarm output signal.

The prototype system needed to be configured with 2oo2 with a safe-voting mechanism for measuring a particular identified vital parametric function. In this system operation approach, both channels A and B received the sample data and computed the vital parameter independently. These measured values were inputs to the safety function, where it correlated and voted to generate the alarm output signal and fault-detection signal, which is as per Table 1 and Figure 5. Furthermore, the generated signals triggered the related diagnostic functions to process the data to select the authentic vital output for display and the fault alarm signal. Thus, this mechanism of configuring and configured safe-voting computation was performed for each essential parameter measured by the medical system.

### 2oo2 Safety-Related Degradation Mechanism

Based on the selected configured parameters in an active system, the system went into a predefined sequence of degradation for any fault detected. These sequences of degradation depended on the configuration of the system and its limitations, such as sensors and availability of the independent processing channels for the same selected parameter. A few identified vital parameters and their feasible degradation scheme are shown in Table 2. Since the system is a modular interface and a provision for high reconfigurability was provided during its initial start phase, it must either have a high-availability system or high safety. Thus, the scheme from 2oo2 to 1oo1 can be altered for high availability with fault-tolerability (or) 2oo2 to 1oo2 to 1oo1 for high-safety with fault-tolerability.


(1) $$\begin{array}{r} Availability=\;\frac{Uptime}{\left(Uptime\;+\;Downtime\right)} \end{array}$$

**Table 2:** 2oo2 configured-system safe degradation scheme.

**Vital sign**	**Fault-tolerant system**	**Degradation level**			**System configuration selection scheme for high availability or safety mode**	
		**1**	**2**	**3**	**High availability and fault-tolerant**	**High safety and fault-tolerant**
Heart rate or pulse	2oo2	1oo2	1oo1	Shut down/Safe mode	2oo2 → 1oo1 → Safe mode	1oo2 → 1oo1 → Safe mode
Respiratory rate	2oo2	1oo2	1oo1	Shut down/Safe mode		
Blood pressure	2oo2	1oo2	1oo1	Shut down/Safe mode		
Body temperature	2oo2	1oo2	1oo1	Shut down/Safe mode		
Pulse oximetry	2oo2	1oo2	1oo1	Shut down/Safe mode		

### Experimental Setup and System Evaluation

The experimental setup and its application protocol detailed in (1) were effectively reused in assessing the proposed concepts. The MATLAB-based CHMS GUI tool was used to configure and capture the resultant data to Plot.

## Experimental Results and Discussion

**Figure 6:**
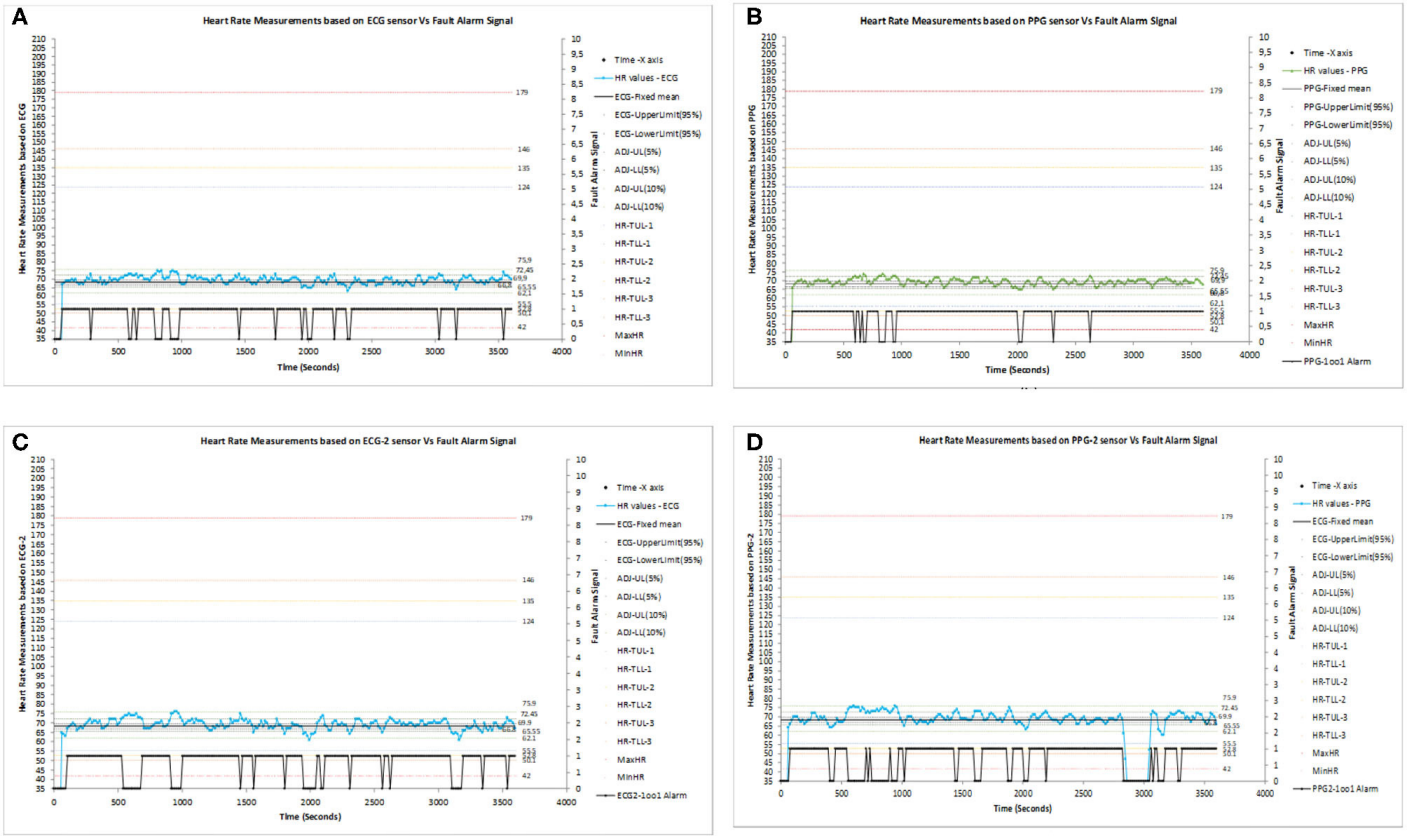
**(A)** Heart rate (HR) measurement based on the ECG sensor vs. the fault alarm signal in 1oo1 configuration. **(B)** HR measurement based on the PPG sensor vs. the fault alarm signal in 1oo1 configuration. **(C)** HR Measurement based on the ECG-2 sensor vs. the fault alarm signal in 1oo1 configuration. **(D)** HR Measurement based on the PPG-2 sensor vs. the fault alarm signal in 1oo1 configuration.

The system configured with 1oo1 could capture HR data values vs. fault alarm signals individually as ECG−1oo1, PPG−1oo1, ECG2–1oo1, and PPG2–1oo1, which are presented in Figures 6A–D from a single subject. The captured alarm data were analyzed to set configurable adjustable upper and lower limits (ADJ-UL and LL) (1).

**Figure 7:**
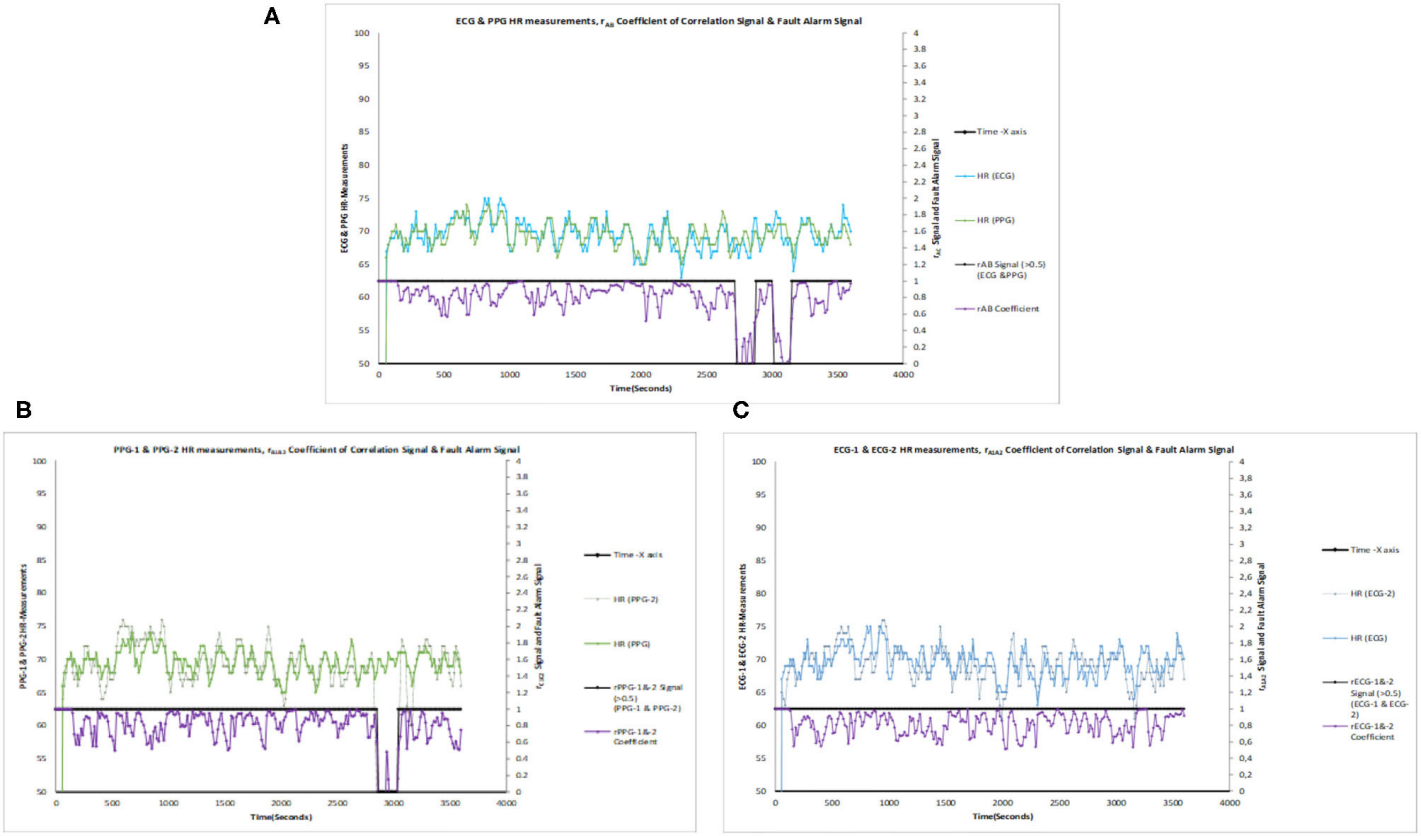
**(A)** ECG and PPG HR measurements, r_AB_ coefficient of correlation signal vs. fault alarm signal. **(B)** ECG-1 and ECG-2 HR measurements, r_A1A2_ coefficient of correlation signal vs. fault alarm signal. **(C)** PPG-1 and PPG-2 HR Measurements, r_B1B2_ coefficient of correlation signal vs. fault alarm signal.

**Figure 8:**
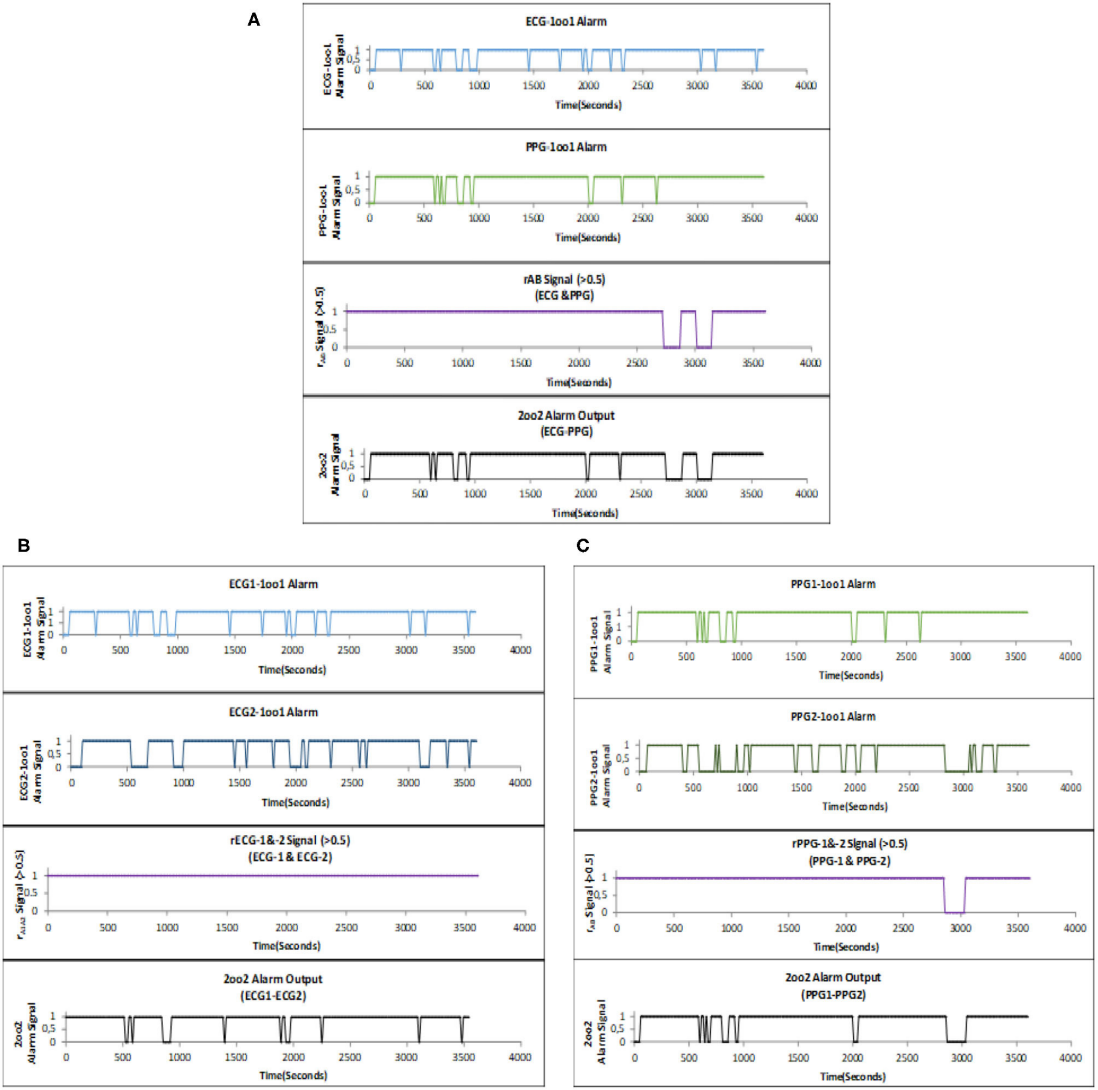
**(A)** ECG–PPG recorded 2oo2 fault alarm output during HR monitoring within a 1-h period. **(B)** ECG1–ECG2 recorded 2oo2 fault alarm output during HR monitoring within a 1-h period. **(C)** PPG1–PPG2 recorded 2oo2 fault alarm output during HR monitoring within a 1-h period.

The configured 2oo2 outputs of ECG–PPG, ECG1–ECG2, and PPG1–PPG2 correlation signal vs. the fault alarm signal are shown in Figures 7A–C. The analysis showed that the reduction of alarm readings from all 50 subjects was similar in meeting the objectives. Figures 8A–C present the configured 2oo2 results of a single subject, which show the reduction in alarms and its related cause-and-effect evaluated analysis presented in Table 3 with inferences.

**Table 3:** 2oo2-based cause and effect—fault detection and evaluation analysis and feasible mitigation solution for the identified cause.

**Evaluation cases**	**Channel-A (ECG)** **(parameter within set limits is true else false)**	**Channel-B (PPG)** **(parameter within set limits is true else false)**	**ECG–PPG** ***r* (>0.5)** **(correlation coefficient)**	**Output signal** **(2oo2 fault-tolerant)** **(true—no alarm; false—alarm)**	**Fault diagnostics required for detected failure yes/no**	**Software functional fault/negation codes** **[Sw, software;** **Ag, algorithm;** **F, function;** **(four-digit)—Code]**	**Fault severity analysis (no-fault/minor/major/critical)** **(algorithms function analysis)**	**Fault description and probable cause**	**Mitigation solution for the identified cause**
Case-1	True	True	True	True	No	SwAgF4001	No-Fault	Data authentic	No action
Case-2	False	True	True	True	Yes	SwAgF4002	Minor	• Data authentic • Software sync/delay issue between channels (or) • Negligible higher pulse count detected in ECG	Perform analytics for consistency (or) repetitive higher pulse count on ecg signal and rectify the issue
Case-3	True	False	True	True	Yes	SwAgF4003	Minor	Similarly, as above for PPG	Similarly, as above for PPG
Case-4	False	False	True	False	No	SwAgF4004	No-Fault	Data authentic	No action
Case-5	True	True	False	False	Yes	SwAgF4005	Major	Possible software sync/Delay issue between channels	Perform analytics to check for the consistent difference between channels to rectify the issue
Case-6	False	True	False	False	Yes	SwAgF4006	Major	Possible cause in software or hardware issue	Perform built-in-test (BIT) and analytics between channels to rectify the issue
Case-7	True	False	False	False	Yes	SwAgF4007	Major	Refer above comment	Refer above comment
Case-8	False	False	False	False	Yes	SwAgF4008	Critical	Possible cause in software or hardware issue	Perform BIT and analytics between channels to rectify the issue

**Figure 9:**
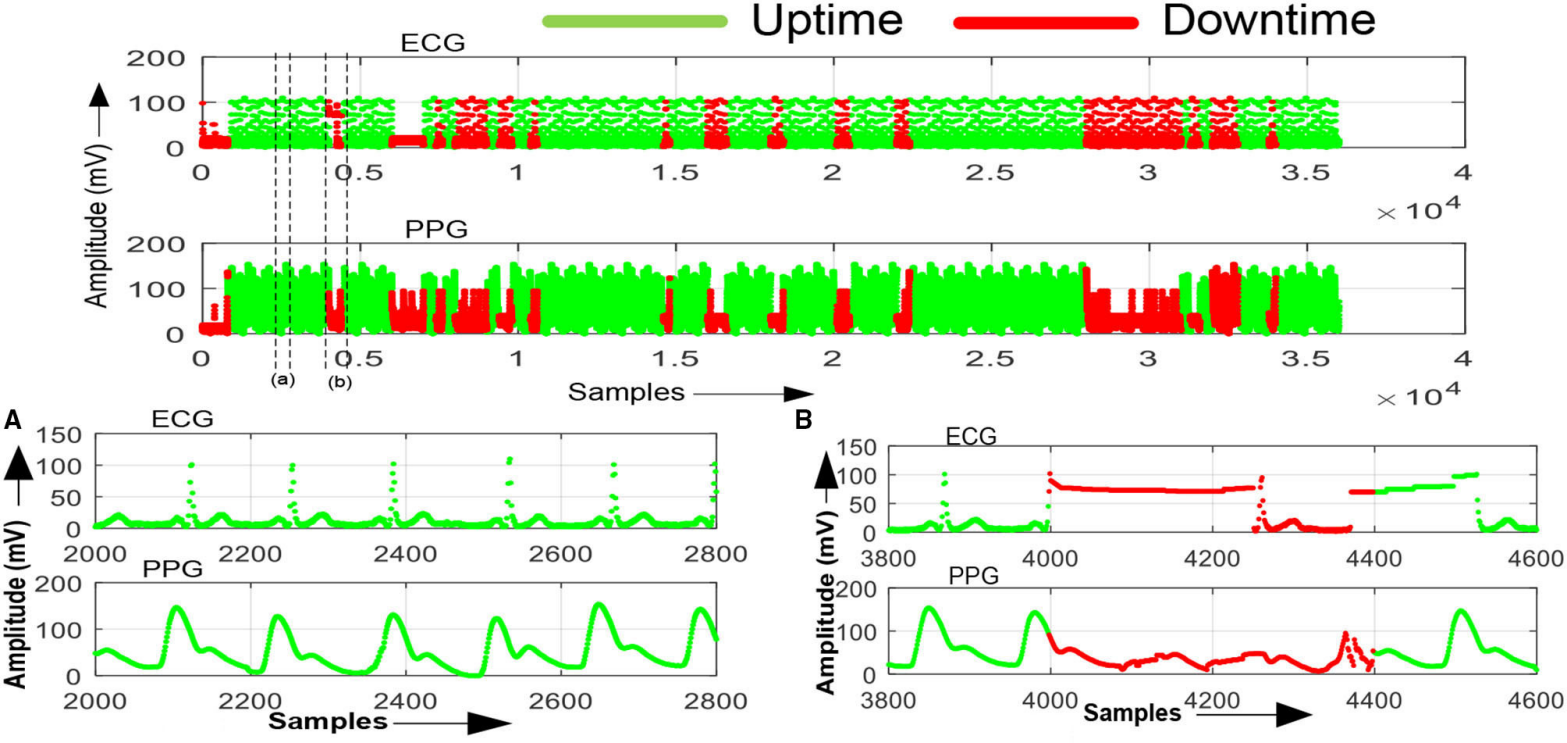
ECG and PPG processed signals data captured using MATLAB tool w.r.t 2oo2 configuration. **(A)** System uptime—normal signal data with no-fault (green). **(B)** System downtime—abnormal signal data with a fault (red).

The sensors and ECG and PPG processed signal data were captured and used to performed analytics with the MATLAB tool by configuring the system in 2oo2 configuration. The tool computed system uptime and downtime by separating the normal and abnormal signal data as shown in Figure 9. Similarly, the data captured for ECG1–ECG2 and PPG1–PPG2 configurations and results were recorded in Table 4 for a single subject. Following Helsinki's declaration and consent, the monitoring system evaluated 50 subjects of various age groups and recorded the uptime and downtime of the system during evaluation, with an average of 50 h for the total operating time of the system and calculated system availability in percentile as per Equation (1). Table 4 provides these results along with system health inferences computed per negation codes specified in Table 3 and assesses in the three configurations that the improvement in system availability significantly improved from 45 to 55%.

**Table 4:** System availability results by configuring the fault-tolerant multimodal sensor system in 2oo2 system configuration mode.

**Fault-tolerant multimodal sensor system configuration**	**Signals**	**Up-time** **(normal Signal)** **(Avg. min)** **(**~**“t”−60 min)**	**Down-timev** **(abnormal Signal)** **(Avg. min)** **(**~**“t”−60 min)**	**System** **availability** **configuration (%)**	**System** **availability** **average of **~**50 h (%)**	**Improvement in system availability using 2oo2 design approach** **% increase (%)**	**Data analytics inferences on system health—common causes**
Concept-1 (ECG–PPG) Evaluation on 50 subjects (1-h/person)	Channel-A (ECG)	53.4	6.6	89	90	57.8	Main hardware causes: • ECG/PCG Sensor probe contact fault. • Power-related faults
	Channel-B (PPG)	55.2	4.8	92%	89		
	Fault-tolerant-output (ECG–PPG)	58.2	1.8	97	95		
Concept-2 (ECG1–ECG2) Evaluation on 50 subjects (1-h/person)	Channel-A (ECG1)	54.6	5.4	91	89	62.5	Main software causes: • Data Sync delay Issues between channels due to algorithm computation times • Inaccuracy in detecting the pulses or due to Noise causes
	Channel-B (ECG2)	56.4	3.6	94	91		
	Fault-tolerant-output (ECG1–ECG2)	58.8	1.2	98	96		
Concept-3 (PPG1–PPG2) Evaluation on 50 subjects (1-h/person)	Channel-A (PPG1)	57.6	2.4	96	91	46.4	
	Channel-B (PPG2)	57.0	3.0	95	94		
	Fault-tolerant-output (PPG1–PPG2)	58.8	1.2	98	97		

However, to validate the improvement function at the system level resilience, some more tests needed to be performed with various sensors to evaluate function performance better. It was observed that, during experimentation, there were limitations such as a signal drift issue during capture and appropriate sync mechanisms needed to improve the signals and channels. The drift of ±2 pulses was noticed as a limitation of the system due to the mediums of the different sensors and the capturing and measurement periods. Furthermore, the system response time of <5 s was noticed as a limitation. Detailed analysis of the uptime period of signals confirmed that the count of truthful pulses in both channels was almost the same during long-term signal capture with negligible or no incorrect pulses and undetected pulses recorded. In contrast, it can mitigate drift issues during short-term signal capture in the design by improving the synchronization mechanism for capturing the signals between the channels.

Further investigation on the uptime and down signals could help the understanding of the causes of various systemic faults within the sensor system. The related fault data captured were analyzed and used to provide inferences in Table 4. Additional analysis of this corresponding prognostic health data was out of the scope of this study as it required defining normal or abnormal vital parameter signal classifications and identifying support mechanisms within the system. In this study, as the focus was on system availability with reduced alarms by processing the signal data through the evaluation of the safety function, only minimal conditions to infer the health of the system were used.

The presented experimental data were captured from each channel with HR measured data. The inverted logic level of the alarm signal was logged for the 1-h duration per subject, with consideration for evaluating the system. The analysis results showed significant improvements in meeting the objectives and a similar systematic approach to further apply this method to other parameter evaluations for safety improvements.

## Conclusion

In this paper, the concept of a fault-tolerant safety-related 2oo2 design approach implemented and evaluated in the configurable medical CHMS, which is a research platform, addressed the effective detection of functional faults, thus improving the uninterruptable function of the targeted medical system by reducing the false or spurious alarms. This framework found a significant reduction in the generation of insignificant alarms and increased uninterruptable system availability by 45–55%. These findings and the design approach were important contributions to issues related to present medical patient monitoring systems without significant impact on cost since it uses the existing system configuration of PPG and ECG signals, along with FPGA technology devices. While eliminating identified issues was specifically focused on, the conceptual design approach may suit medical monitoring systems, implying that the findings are likely to be of importance to the design of medical monitoring and control systems. In terms of future research, it is particularly suggested to use diverse algorithms and sensors or evaluation with a combination of these with effective predictive system maintenance, which could help eliminate spurious alarms with a reduced downtime of the system and produce more accurate data vital parameters.

## Data Availability Statement

The datasets presented in this article are not readily available because Non Disclosure Agreement with the patients. Requests to access the datasets should be directed to Prasadraju Lakkamraju, prasadraju.lvr@gmail.com.

## Ethics Statement

The studies involving human participants were reviewed and approved by Citizens Hospitals Hyderabad. The patients/participants provided their written informed consent to participate in this study.

## Author Contributions

All authors listed have made a substantial, direct and intellectual contribution to the work, and approved it for publication.

## Conflict of Interest

The authors declare that the research was conducted in the absence of any commercial or financial relationships that could be construed as a potential conflict of interest.

## Publisher's Note

All claims expressed in this article are solely those of the authors and do not necessarily represent those of their affiliated organizations, or those of the publisher, the editors and the reviewers. Any product that may be evaluated in this article, or claim that may be made by its manufacturer, is not guaranteed or endorsed by the publisher.
